# Minimal versus Intensive: How the Pruning Intensity Affects Occurrence of Grapevine Leaf Stripe Disease, Wood Integrity, and the Mycobiome in Grapevine Trunks

**DOI:** 10.3390/jof8030247

**Published:** 2022-02-28

**Authors:** Christian Kraus, Carolin Rauch, Elisa Maria Kalvelage, Falk Hubertus Behrens, Dagmar d’Aguiar, Cornelia Dubois, Michael Fischer

**Affiliations:** Julius Kühn-Institute, Federal Research Centre of Cultivated Plants, Plant Protection in Fruit Crops and Viticulture, 76833 Siebeldingen, Germany; carolin.rauch@julius-kuehn.de (C.R.); elisa.kalvelage@julius-kuehn.de (E.M.K.); falk.behrens@julius-kuehn.de (F.H.B.); dagmar.daguiar@julius-kuehn.de (D.d.); cornelia.dubois@julius-kuehn.de (C.D.); michael.fischer@julius-kuehn.de (M.F.)

**Keywords:** esca, grapevine, pruning, grapevine-trunk disease, monitoring, SMPH, VSP, mycobiome

## Abstract

Previous works on grapevine-trunk diseases indicate that minimal or non-pruning of the grapevine under certain circumstances can significantly reduce the risk of symptom expression. Nevertheless, knowledge of the mechanisms behind these observations are limited. Therefore, it was the aim of this study to investigate in more detail the effect of pruning intensity on the grapevine trunk by means of trunk integrity and the fungal community in the wood tissue. Two German vineyards partially trained in vertical-shoot position and semi-minimally pruned hedges were chosen for this survey due to the accessibility of multi-annual esca-monitoring data. The results revealed that only in one of the two vineyards was the incidence of external esca symptoms significantly reduced over a period of five years (2017–2021) by minimal pruning, which was up to 73.7% compared to intensive pruning. In both vineyards, the trunks of intensively pruned vines not only had more pruning wounds on the trunk (by 86.0% and 72.9%, respectively) than minimally pruned vines, but also exhibited a larger (by 19.3% and 14.7%, respectively) circumference of the trunk head. In addition, the percentage of white rot and necrosis in the trunks of esca-positive and esca-negative vines was analyzed and compared between the two pruning intensities; hereby, significant differences were only found for esca-negative ‘Dornfelder’ vines, in which the proportion of necrosis was higher for intensively pruned vines (23.0%) than for minimally pruned vines (11.5%). The fungal communities of the differently pruned vine trunks were mainly dominated by four genera, which are also associated with GTDs: *Diplodia*, *Eutypa*, *Fomitiporia* and *Phaeomoniella*. All in all, the fungal diversity and community composition did not differ between minimally and intensively pruned, esca-positive vines.

## 1. Introduction

The esca complex is one of three major grapevine-trunk diseases (GTDs), which are a group of destructive wood diseases that cause massive losses every year in vine-growing regions all over the world [1,2]. The causal agents of GTDs are phytopathogenic fungi that invade and colonize the vascular system of the vines’ wood tissue, thereby causing symptoms in the trunk, leaves and berries, probably by the secretion of extracellular compounds and/or vessel occlusion [3,4,5,6]. However, the symptomology of the GTDs is as diverse as the fungal pathogens involved. Eutypa dieback, which is characterized by stunted shoots and chlorotic leaves, is caused by fungi of the family Diatrypaceae, with *Eutypa lata* being the most virulent and common [7,8,9,10]. Members of the Botryosphaeriaceae, e.g., *Botryosphaeria*, *Diplodia* or *Neofusicoccum*, are associated with Botrysphaeria dieback [11,12]. Here, the affected vines exhibit shoot dieback, perennial cancer and vascular discoloration of the wood [13]. For the esca complex, five different disease/syndrome classes were determined by means of vine age, symptom type and the fungi involved: (1) dark wood streaking and (2) Petri disease in young vines (<7 years); (3) white rot and (4) esca proper in old vines (>8 years); (5) grapevine-leaf-stripe disease (GLSD), which is often associated with partial or complete apoplexy, to be found in vines of all ages [2,5,14,15,16]. *Phaeomoniella chlamydospora*, *Phaeoacremonium* spp. and *Cadophora* spp. are frequently found in young, esca-affected vines [17,18,19,20,21,22]. In older vines, this set of fungi are also common; however, this is in combination with basidiomycetes, mainly from the genus *Fomitiporia* spp., which is the causal agent of white rot [23,24,25,26,27]. Besides the already-named fungal genera and species, the list of fungi associated with the esca complex has become even longer over recent decades and is still increasing [28,29,30,31,32,33].

Although pathogenic fungi play a key role within the esca complex, there are several other biotic and abiotic factors that drive this longsome disease process [34,35]. For instance, the incidence of external esca symptoms, i.e., GLSD, mainly depends on plant age; the older the vines become, the higher the chance of expressing symptoms [19,36,37,38]. Furthermore, it has been shown that some cultivars, e.g., ‘Cabernet Sauvignon’, ‘Grechetto’ or ‘Sangiovese’, are more susceptible to GLSD than others, e.g., ‘Chardonnay’, ‘Pinot’ or ‘Merlot’ [39,40,41,42]. This cultivar-dependent susceptibility probably correlates with the number and the width of xylem vessels; cultivars with larger vessels are considered to be more vulnerable to drought-stress-induced xylem cavitation and embolism [4,6,43,44,45,46]. In this context, drought-stress or, in general, climate conditions have also been reported to contribute to symptom expression [34,47,48,49,50,51]. Overall, the esca-disease complex is driven by a complex network of multiple known and yet unknown influencing factors that may interact with each other.

**Figure 1:**
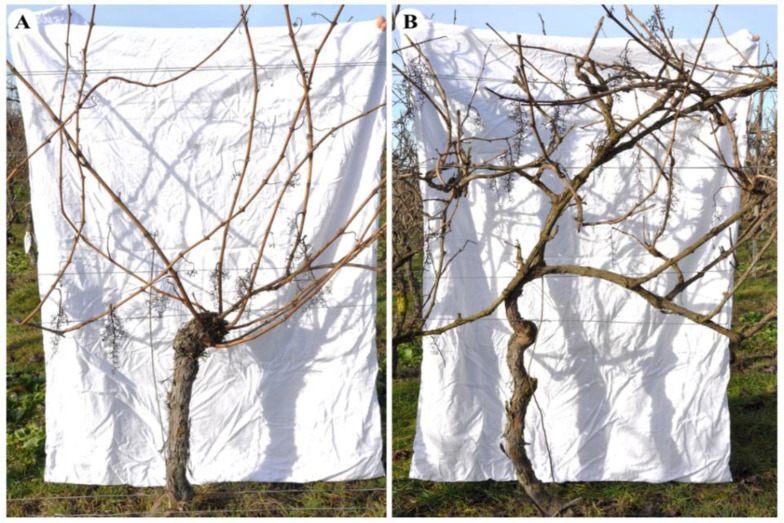
Grapevine cv. ‘Dornfelder’ trained in (**A**) vertical-shoot position (VSP) and (**B**) semi-minimally pruned hedge (SMPH) at dormancy and before winter pruning.

Another key driver frequently mentioned in the context of GTD management may lie in the pruning scheme as part of the grapevine-training system [1,52]. Since pruning wounds are supposed to be the main gate for fungal infections of the grapevine vascular system, intensive pruning schemes, e.g., cane pruning or vertical-shoot position (VSP, Figure 1), might enhance disease incidence, while non- or minimal-pruning schemes, e.g., semi-minimally pruned hedge (SMPH), might reduce this risk [38,53]. With this background, an esca assessment conducted in German vineyards in 2009 showed higher GLSD incidence in VSP-trained vines than in SMPH-trained vines [54]. A comparison of spur and minimal pruning in two French vineyards that were planted with the cultivars ‘Syrah’ and ‘Mourvèdre’ showed similar results: the incidence was higher for spur-pruned vines compared to minimally pruned vines [55]. In addition, Lecomte et al. [56] demonstrated a reduction in GLSD-affected vines in non-pruned vineyards compared to ‘Guyot-Lépine’ and cordon vineyards, respectively. Recently, we presented data from a four-year survey (2015–2018) on GLSD performed in 12 vineyards that were partially trained in VSP and SMPH [51]. The results of GLSD incidence between the two pruning systems were inconsistent over the four years: in 2015, no differences were found; in 2016, the incidence was higher for SMPH compared to VSP; and in the last two years the opposite was the case, with VSP vines showing more symptoms. It was assumed that the complexity of the disease and the inherent vineyard diversity, i.e., different cultivars, location, age, etc., eventually led to the heterogeneous results.

Although the assessments demonstrated significant differences regarding the susceptibility of intensively and minimally pruned vines against esca, data on the possible causes for these findings are rare. Therefore, it was the aim of this study to compare in more detail intensively and minimally pruned vines. Two German vineyards planted with VSP and SMPH vines were chosen for this study due to the multi-annual esca-monitoring data available. In these vineyards, the trunk morphologies of the two different pruning intensities were compared by means of pruning-wound quantity and size, as well as by trunk dimension, i.e., height, head and waist circumference. Furthermore, the inner-wood integrity of VSP and SMPH vines was assessed by measuring the relative amount of white rot and wood discoloration. Finally, the mycobiome in esca-affected intensively and minimally pruned vines was analyzed using a cultivation-independent method (next-generation sequencing). The obtained results may help to better understand in which way the pruning system influences the incidence of GLSD. In addition, the data collected in this study should help to develop pruning or training strategies that reduce the incidence of GLSD in vineyards.

## 2. Materials and Methods

### 2.1. Sample Sites and Plant Material

The vineyards chosen for this survey were located in the north of Rhineland-Palatinate, Germany: one younger vineyard that was planted in 2003 with the cultivar ‘Dornfelder’ and one older vineyard that was planted in 1984 with the cultivar ‘Müller-Thurgau’. The vineyards were about 28 km apart from another and were established on a plain surface with loamy soil. In both vineyards, the plants were originally trained in VSP and partially converted into SMPH in 2013 (‘Dornfelder’) and 2008 (‘Müller-Thurgau’). In 2020, 726 additional VSP vines in the ‘Dornfelder’ vineyard were converted into SMPH to study the effect of late pruning conversion, i.e., changing from intensive to minimal pruning in an advanced life stage (17 years) of the vineyard, on the incidence of esca. Further vineyard characteristics are shown in Table 1.

For VSP, the vines were pruned by hand at dormancy and old canes from the preseason were removed. One remaining, annual cane was horizontally attached to the trellis. The SMPH vines were roughly pruned with a mechanical trimmer on the sides and on top. As a result, the plants had a height of about 2 m and a canopy width of about 50 cm. The mechanical trimmer was also used for a summer pruning, which was performed on both pruning systems, VSP and SMPH.

### 2.2. Monitoring

From 2015 to 2020 the ‘Müller-Thurgau’ vineyard was annually monitored for external esca symptoms, i.e., GLSD (“tiger-strips”) and apoplexy. Monitoring took place five times in a season from the beginning of July until the beginning of September in a two-week rhythm. Esca-affected plants were marked in a field map. The ‘Dornfelder’ vineyard was monitored the same way from 2017 to 2021.

For statistical analysis of the esca incidence in the two pruning systems, Fisher’s exact test was applied in RStudio [57] using the absolute numbers of esca-symptomatic (detected until September) and asymptomatic vines.

### 2.3. Grapevine Trunk Properties

The number of pruning wounds and combined wound sizes were determined in order to compare the impact on the grapevine trunk caused by the pruning systems. In addition, the trunk height, the circumference of the trunk head and the circumference of the trunk center (middle section) was measured. For the measurements, 20 vines per pruning system and study site were randomly picked. The obtained data were evaluated by using the program RStudio [57] and Student’s *t*-test.

### 2.4. Grapevine Wood Integrity

A total of 30 grapevine trunks per pruning system were removed from each study site by cutting the vines below the graft union. From these, 15 showed GLSD symptoms and 15 were asymptomatic during the whole survey. The trunks were brought to the lab for further processing and examination.

With a band saw, the trunks were longitudinally cut into two halves and the inner view of both halves was documented with a Nikon digital camera D90. The pictures were processed by hand with the program paint.net v4.1.5 as follows: The background was erased, wood showing white rot was colored in green and discolored/necrotic wood was highlighted in blue. Afterwards the area size of white-rot and necrotic-wood tissue in relation to healthy wood was determined by using ImageJ version 1.8.0_172 [58]. Statistics were analyzed using RStudio [57] and two-way ANOVA.

### 2.5. Local Climate

Weather conditions, especially the amount of rainfall, may play a significant role in the esca-disease process. Therefore, precipitation data at the two vineyard locations were obtained from weather stations in proximity to the study sites, which are run by the DLR Rhineland-Palatinate (www.dlr.rlp.de, accessed on 10 January 2022).

### 2.6. Wood-Sample Preparation and DNA Extraction

From each vineyard (‘Dornfelder’ and ‘Müller-Thurgau’) and pruning system (VSP and SMPH), nine grapevine trunks (all positive for GLSD) were collected by sawing the vine below the graft union. From the trunks, wood samples were taken using a core drill with a diameter of 12 mm and 300 mm length. For each vine, three holes were drilled into the trunk head and three holes into the center of the trunk. The core samples from each vine and trunk position were collected in a sterile plastic bag, resulting in a sample size of 72 (two vineyards, two pruning systems, two trunk positions). Samples were brought to the lab for further processing.

Under sterile laminar airflow, the samples were surface disinfected by successive submersion in 2.5% sodium hypochlorite, 70% ethanol and twice in sterile distilled water, each for 30 s [59]. Afterwards, the wood samples were cut into smaller pieces of about 25 mm^3^ in size, collected in a 50 mL falcon tube and stored at −20 °C.

For the mycobiome analysis, a cultivation-independent method (next-generation sequencing) was chosen. For this, the gDNA from the wood samples was extracted using the cetyltrimethylammonium bromide (CTAB)/β-mercaptoethanol method [52]. Prior to the extraction process, the frozen wood pieces were pulverized with a heavy steel ball and a tissue lyser (Tissue Lyser 2, Qiagen, Hilden, Germany) by intensive shaking at 30,000 Hz for 2 min. The gDNA was extracted from 200 mg of wood powder. The quality and quantity of the obtained gDNA was analyzed with a spectrophotometer (Nanodrop 2000c, Thermo Fisher Scientific, Waltham, MA, USA) and visually by running a 2% agarose gel. Finally, the gDNA concentration was adjusted to 10 ng/mL.

### 2.7. Next-Generation Sequencing (NGS)

The gDNA samples were shipped to Microsynth AG (Next-Generation Sequencing Department, Balgach, Switzerland) for metabarcoding analysis. For library preparation, the ITS2 region was amplified with a Nextera two-step PCR using the primer pair ITS3 and ITS4 [60]. The amplicons were then purified and pooled. Sequencing took place on an Illumina MiSeq (v2, micro, 2 × 250 bp; San Diego, CA, USA).

### 2.8. Mycobiome—Raw Data Processing

Metabarcoding raw-data processing was conducted by an amplicon-denoising workflow based on the R package dada2 [61]. Primers were clipped using Cutadapt v3.4 [62] and the R package ShortRead [63]. N-filtered and clipped raw reads were quality filtered with the maximum expected errors set to two and the truncation of reads at a quality score of ten or below. Forward and reverse reads were denoised with independent sample inference, merged allowing for no mismatches, purged from chimeric sequences and taxonomically assigned using the naive Bayesian classifier method [64] on the UNITE v8.2 database [65] with a minimum bootstrapping support of 70. Raw sequence data was deposited into the NCBI SRA database (https://www.ncbi.nlm.nih.gov/sra, accessed on 10 January 2022) with accession number PRJNA796210.

### 2.9. Mycobiome—Statistical Analysis

The statistical analyses of the mycobiome data were all performed using RStudio software [57] and the package vegan [66]. Mean read counts of 100 rarefactions to the lowest sequencing depth were used for all analyses. Differences in alpha diversity between the two pruning systems were calculated using Wilcoxon rank-sum test. A principal-coordinates analysis (PCoA) based on Bray–Curtis dissimilarities was performed for each vineyard and grouped by the pruning system and trunk location. Furthermore, a PERMANOVA and ANOSIM were run to check for differences in the fungal-community composition between intensive and minimal pruning. Student’s *t*-test was chosen to find significant differences in the relative abundance of the four selected, GTD-associated fungal genera (*Diplodia*, *Eutypa*, *Fomitiporia*, *Phaeomoniella*) when comparing the mycobiome of VSP and SMPH vines. Here, the relative abundance is defined as the number of reads of one genus relative to the total number of reads in one sample.

## 3. Results

### 3.1. Esca Incidence

**Figure 2:**
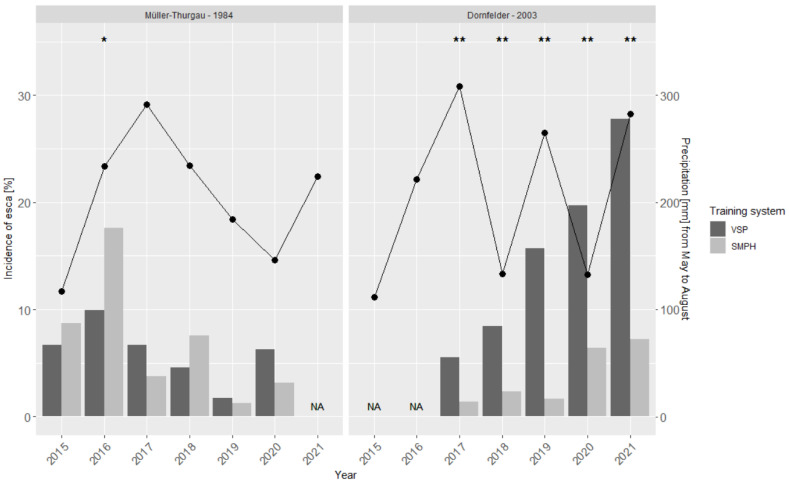
Total incidence of plants [%] showing external esca symptoms, i.e., GLSD (“tiger-stripes”) and apoplexy in the two vineyards (‘Dornfelder’ and ‘Müller-Thurgau’) as a function of the training system (VSP and SMPH). Black line indicates the total amount of rain [mm] from May to August for each year. NA = data not available. Asterisks indicate significant differences regarding esca incidence between the training systems according to Fisher’s exact test (* *p* < 0.05, ** *p* < 0.001).

The esca monitoring produced inconclusive results in the two vineyards, ‘Dornfelder’ and ‘Müller-Thurgau’ (Figure 2). In the latter, only in 2016 were significant differences between the pruning systems found, when the SMPH vines (17.6%) expressed more external esca symptoms than the VSP vines (10%). These were the highest noted incidences in this vineyard during the six years of monitoring. The lowest incidences in the ‘Müller-Thurgau’ vineyard were found in 2019 with only 1.7% of symptomatic VSP vines and 1.3% of SMPH vines. No correlation was found between the amount of rain during the season (May to August) and the annual esca incidence. The same is true for the ‘Dornfelder’ vineyard; years with increased precipitation were not correlated with a higher or lower incidence of esca. During the five-year monitoring of the ‘Dornfelder’ vineyard, the incidence of esca in the VSP-trained section exponentially increased from 5.5% in 2017 to 27.8% in 2021. During this period, the incidence of esca in the SMPH-trained section in the period 2017–2019 did not exceed 2.3%, while in 2020–2021 the rate reached 6.3% and 7.3%, respectively. Comparing the numbers of (a)symptomatic vines in the VSP and SMPH sections resulted in significant differences for all years, with VSP vines being more symptomatic than SMPH vines.

**Figure 3:**
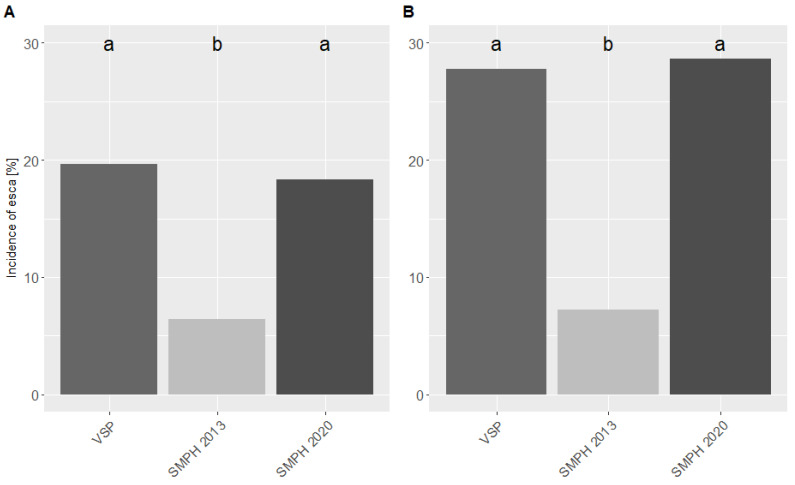
Total incidence of plants [%] showing external esca symptoms, i.e., GLSD (“tiger-stripes”) and apoplexy in the ‘Dornfelder’ vineyard monitored in (**A**) 2020 and (**B**) 2021 as a function of the pruning system (VSP and SMPH) and the time period of pruning conversion (‘2013’ = after 10 years of intensive pruning; ‘2020’ = after 17 years of intensive pruning). Different letters indicate significant differences in esca incidence according to Fisher’s exact test (*p* < 0.001).

In 2020, additional VSP-trained vines (726) were converted into SMPH in the ‘Dornfelder’ vineyard. The esca monitoring in 2020 revealed an equal rate of symptomatic VSP vines (19.7%) and recently converted SMPH vines (18.3%; Figure 3). The SMPH vines, which were converted seven years ago, had a lower incidence of 6.4%. Additionally, in 2021, no significant differences were noted between VSP vines (27.8%) and SMPH vines (28.7%), the latter having been minimally pruned for two years by then. The incidence of vines minimally pruned for eight years was significantly lower (7.2%) compared to the other two variants.

### 3.2. Grapevine Trunk Integrity

Five properties were chosen in order to study the effect of pruning intensity on the grapevine trunk (Table 2). (1) In both vineyards, the number of pruning wounds was significantly higher for the VSP vines compared to the SMPH vines. Intensively pruned vines from the ‘Dornfelder’ vineyard showed 17.3 ± 2.9 pruning wounds, while minimally pruned vines had only 9.3 ± 2.8 wounds. In the ‘Müller-Thurgau’ field, 23.0 ± 6.3 wounds were found on the VSP vines and 13.3. ± 3.4 wounds on the SMPH vines. (2) The mean size of the pruning wounds was the same for both pruning systems and vineyards, and so was the (3) trunk height. Regarding the circumference of the (4) trunk head and (5) middle section, significant differences between the pruning systems were only found for the trunk head. Here, the mean circumference of the VSP vines was about 16.2% (‘Dornfelder’ vineyard) and 12.4% (‘Müller-Thurgau’ vineyard), respectively, which was larger compared to the SMPH vines. The circumference of the trunk’s middle section was not affected by the pruning system, neither for ‘Dornfelder’ vines nor for ‘Müller-Thurgau’ vines.

Besides the above-mentioned grapevine-trunk properties, the inner-wood integrity of intensively and minimally pruned grapevines was compared by evaluating the relative area size of white rot and necrosis in the longitudinal sections. The analysis was performed with respect to the esca status of the examined grapevines, i.e., with or without external symptoms.

**Figure 4:**
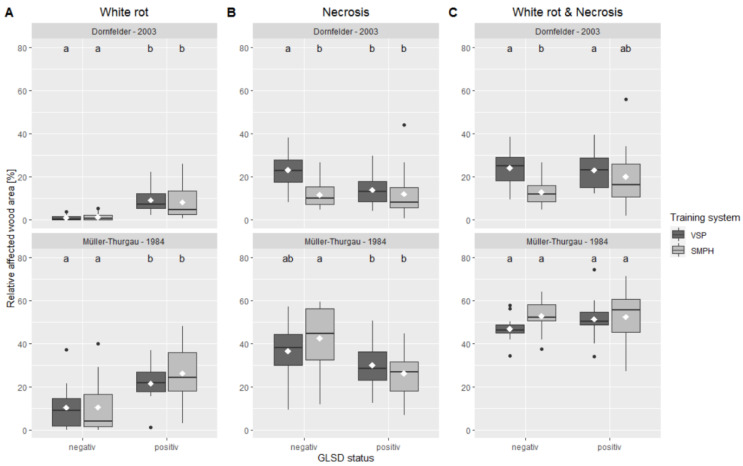
Inner-wood integrity by means of (**A**) white rot, (**B**) discoloration/necrosis and (**C**) both combined in terms of percentage of affected area in longitudinal sections as a function of the training system (VSP and SMPH) and esca status (‘positive’ and ‘negative’) in the two studied vineyards (‘Dornfelder’ and ‘Müller-Thurgau’). Different letters indicate significant differences according to two-way ANOVA (*p* < 0.05).

Regarding white rot, esca-positive vines showed a higher proportion of symptomatic wood than esca-negative vines, irrespective of the pruning system and vineyard (Figure 4). In the ‘Dornfelder’ vineyard, the mean percentage of the white-rot area was 0.9% (VSP) and 1.2% (SMPH) for vines without symptoms and 9.1% (VSP) and 8.0% (SMPH) for esca-affected vines. Compared to the ‘Dornfelder’ vineyard, the proportion of white rot in the trunk was about ten times (esca negative) and about 2.5 times (esca positive) higher in the ‘Müller-Thurgau’ vineyard. Here, 10.3% (VSP) and 10.4% (SMPH) of the wood was symptomatic in esca-negative vines and 21.4% (VSP) and 26.1% (SMPH) in esca-positive vines.

The results of the wood-necrosis assessment were not in accordance with the white-rot assessment in terms of the esca status. Both VSP- and SMPH-trained and esca-negative ‘Müller-Thurgau’ vines showed a higher mean necrotic area with 36.4% and 42.4%, respectively, than the esca-positive ‘Müller-Thurgau’ vines (VSP = 29.9%; SMPH = 26.2%). For the ‘Dornfelder’ vineyard, the mean necrotic area in esca-negative VSP vine was significantly higher (23.0%) compared to esca-positive VSP (13.8%), SMPH (11.9%), and esca-negative SMPH vines (11.5%).

When combining white rot and dark necrosis, the mean area of the affected wood is higher for the intensively pruned (esca negative) ‘Dornfelder’ vines than for the minimally pruned (esca positive) ‘Dornfelder’ vines. However, in the ‘Müller-Thurgau’ vineyard, no differences were found irrespective of esca status or training system.

### 3.3. Mycobiome in the Trunk

**Figure 5:**
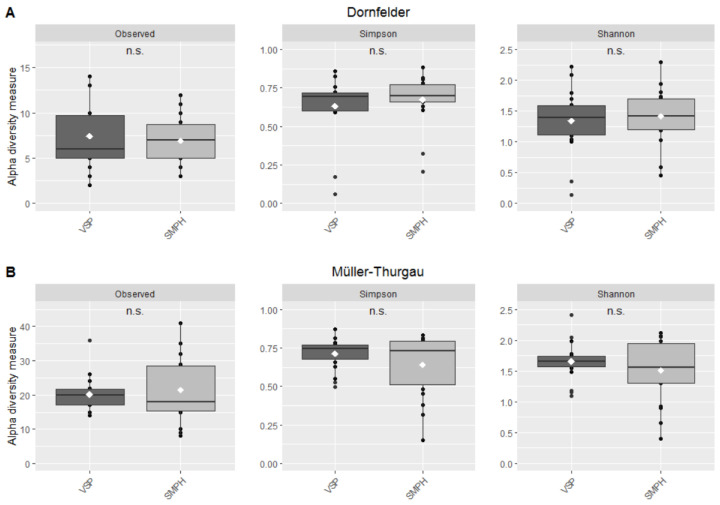
Fungal alpha diversity, i.e., observed OTUs, Simpson and Shannon indices in the grapevine trunk as a function of the training system (VSP and SMPH), for samples collected in the (**A**) ‘Dornfelder’ and (**B**) ‘Müller-Thurgau’ vineyards.

In total 525 fungal operational-taxonomic units (OTUs) were identified from 72 grapevine wood samples by metabarcoding of the ITS2 region (ITS3-ITS4). After rarefaction, 111 OTUs were left in the ‘Dornfelder’ samples and 267 OTUs in the ‘Müller-Thurgau’ samples. The mean number of observed OTUs was about three times higher in the ‘Müller-Thurgau’ samples (VSP = 20.2, SMPH = 21.4) compared to the ‘Dornfelder’ samples (VSP = 7.4, SMPH = 6.9; Figure 5). However, for both vineyards, no differences were found in the observed OTUs between the two training systems. An analysis of the alpha diversity also revealed no differences between intensive and minimal pruning; for the ‘Dornfelder’ samples, the Simpson index was 0.63 for VSP and 0.67 for SMPH, while the Shannon index was 1.34 for VSP and 1.41 for SMPH. The samples taken from ‘Müller-Thurgau’ vines had a Simpson index of 0.71 (VSP) and 0.64 (SMPH). The Shannon indices were 1.65 (VSP) and 1.51 (SMPH).

**Figure 6:**
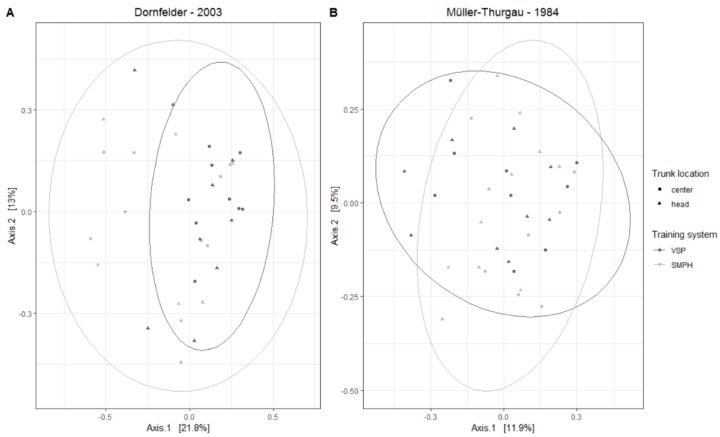
PCoA based on Bray–Curtis dissimilarities of the wood samples collected from the (**A**) ‘Dornfelder’ and (**B**) ‘Müller-Thurgau’ vineyards. Samples were grouped by origin, i.e., trunk center (●) and trunk head (▲), and training system, i.e., VSP (dark) and SMPH (bright). Ellipses represent 95% confidence intervals for the mycobiome from each group (training system).

A PCoA of the wood samples collected from two different trunk origins (center and head) of VSP and SMPH vines (esca symptomatic) was performed to find possible differences in the fungal-community composition (Figure 6). The results revealed a high overlap of the fungal communities from VSP and SMPH vines in the ‘Dornfelder’ (ANOSIM: R = 0.09405, *p* = 0.0132; PERMANOVA: F = 0.797, *p* = 0.386) as well as in the ‘Müller-Thurgau’ vineyard (ANOSIM: R = 0.07303, *p* = 0.0551; PERMANOVA: F = 2.9117, *p* = 0.103), indicating no influence of the pruning intensity on the mycobiome composition in the grapevine trunk.

**Figure 7:**
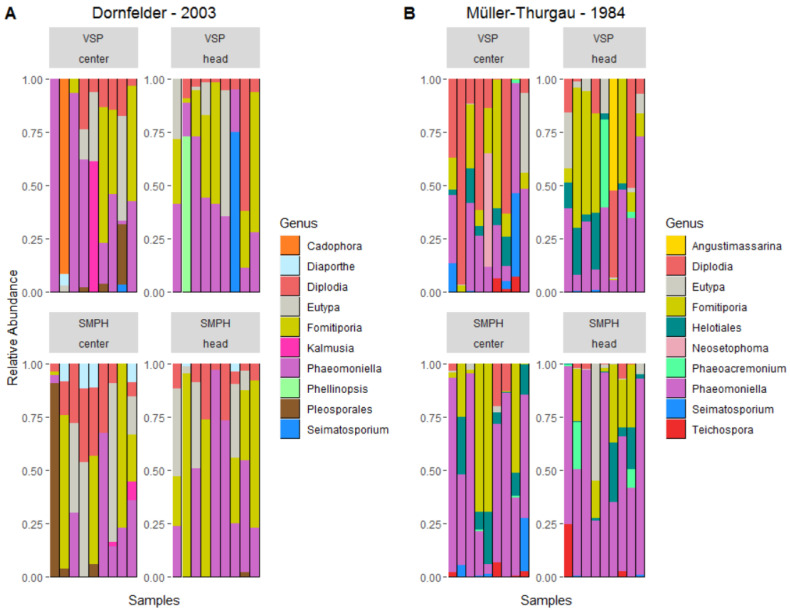
Relative abundance of the top ten genera, which are not assigned to ‘Unknown’, found in the wood samples collected from the (**A**) ‘Dornfelder’ and (**B**) ‘Müller-Thurgau’ vineyards. Samples were grouped by training system (VSP and SMPH) and origin (trunk center and trunk head).

**Figure A1:**
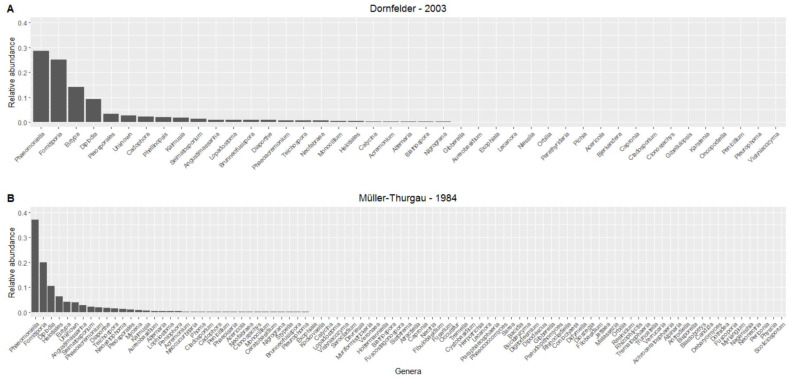
Overall relative abundance of the fungal genera identified by metabarcoding of 72 grapevine wood samples taken from (**A**) ‘Dornfelder’ and (**B**) ‘Müller-Thurgau’ vineyards, respectively.

Wood samples collected from ‘Müller-Thurgau’ vines contained 111 OTUs and were assigned to 43 genera. The ten most abundantly identified genera/orders, which are not assigned to ‘Unknown’, covered 85% of all reads (Figure 7 and Figure A1): *Phaeomoniella* (29%), *Fomitiporia* (23%), *Eutypa* (13%), *Diplodia* (9%), *Pleosporales* (3%), *Cadophora* (2%), *Phellinopsis* (2%), *Kalmusia* (2%), *Seimatosporium* (1%) and *Lopadostoma* (1%). Samples collected from ‘Dornfelder’ vines contained 267 OTUs, which were assigned to 95 genera. Here, the ten most abundant genera/order covered 84% of all reads: *Phaeomoniella* (37%), *Fomitiporia* (19%), *Diplodia* (11%), *Helotiales* (6%), *Eutypa* (4%), *Seimatosporium* (2%), *Phaeoacremonium* (2%), *Neosetophoma* (1%), *Teichospora* (1%) and *Angustimassarina* (1%).

**Figure 8:**
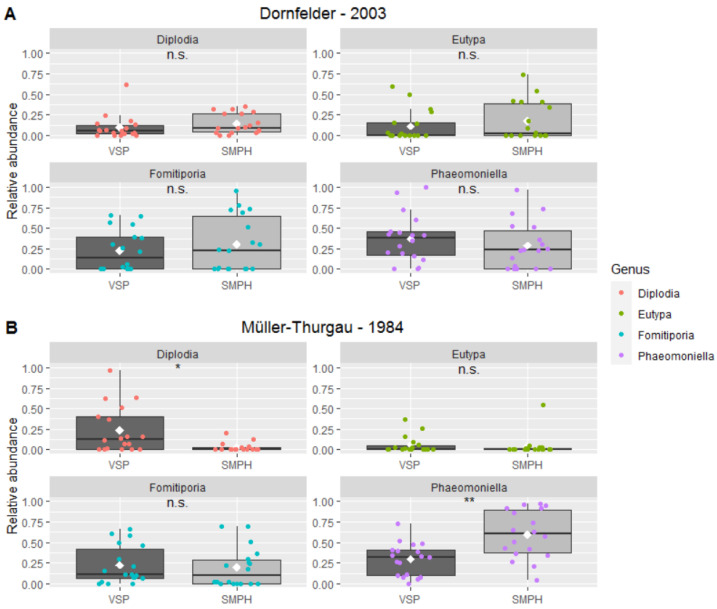
Relative abundance of four most common fungal genera associated with GTD in wood samples from VSP- and SMPH-trained vines collected from the (**A**) ‘Dornfelder’ and (**B**) ‘Müller-Thurgau’ vineyard. Asterisks indicate significant differences according to Student’s *t*-Test (* *p* < 0.05, ** *p* < 0.001).

Four fungal genera, all associated with GTDs, dominate the fungal mycobiome in the collected wood samples: *Diplodia* (Botryosphaeria dieback), *Eutypa* (Eutypa dieback), *Fomitiporia* (esca) and *Phaeomoniella* (esca). For the ‘Dornfelder’ samples, their relative abundance is not different when comparing intensive and minimal pruning (Figure 8). However, for the ‘Müller-Thurgau’ samples, *Diplodia* was about ten times more abundant in the VSP samples (23.6%) than in the SMPH samples (2.8%). Furthermore, the abundance of *Phaeomoniella* was about twice as high in the SMPH samples (59.8%) compared to the VSP samples (29.5%).

## 4. Discussion

Pruning intensity can have an impact on the incidence of external esca symptoms in the grapevine trunk. While intensive pruning may favor symptom occurrence, minimal pruning may reduce the risk [38,51,54,55,56]. However, this seems to be true only under certain circumstances, and one factor could be the time that minimal pruning is started. In the ‘Müller-Thurgau’ vineyard, where a five-year survey found almost no differences in esca incidence between VSP and SMPH vines, minimal pruning was started after 24 years of intensive pruning. On the other hand, in the ‘Dornfelder’ vineyard, the number of esca-affected vines was significantly higher for the VSP vines compared to the SMPH vines throughout the six years of assessment. Here, the conversion to minimal pruning started earlier, i.e., after ten years of intensive pruning. The assumption that only premature minimal pruning can significantly reduce esca occurrence is supported by data collected from two vineyards in France, planted in 1999 (‘Mourvèdre’) and 1994 (‘Syrah’; 55). Four and eight years, respectively, after planting, half of the vines were converted from spur pruning to minimal pruning. Esca monitoring in these vineyards that was conducted in 2012 revealed a higher incidence for spur-pruned (32% and 46%, respectively) vines compared to minimally pruned vines (12% and 16%, respectively). On the other hand, a four-year survey conducted in German vineyards showed no clear differences between VSP and SMPH vines in older vineyards (>27 years) with a late pruning conversion (>20 years after planting; 51). After 17 years of intensive pruning, a late conversion to minimal pruning, as performed in the ‘Dornfelder’ vineyard, could not reduce the incidence of external esca symptoms. This suggests that in order to considerably decrease the risk of esca by minimal pruning, the conversion should be performed as early as possible, preferably before the 10th year of standing.

The reason why minimal pruning under certain conditions can minimize the esca risk is most likely due to the reduced number of pruning wounds on the trunk. As expected, fewer pruning wounds were found on the SMPH vines than on VSP vines. Moreover, intensive pruning seems to increase the trunk’s head circumference; the heads of the VSP vines were 17% (‘Dornfelder’) and 12% (‘Müller-Thurgau’) larger compared to SMPH vines, respectively. Removing shoots from the trunk head is a stressful situation for vines; the wound has to be closed, new shoots formed and therefore the vascular system needs to be restructured. This probably leads to additional wood formation and callusing in the trunk’s head, thereby increasing the circumference. The annual restructuring of the vascular system caused by intensive pruning also provokes disturbance in the sap flow and induces occlusion, both of which are associated with external esca symptoms, i.e., GLSD and apoplexy [6,67,68]. However, for minimally pruned vines, omitting intensive head pruning can minimize restructuring and maintain vessel integrity. In the case of the ‘Müller-Thurgau’ vineyard, where pruning conversion took place in an advanced life stage of the vineyard, the vascular system was permanently disrupted after 24 years of intensive pruning, which could explain why minimal pruning in such vineyards does not necessarily lower the incidence of esca.

Besides vessel restructuring by the plant, pathogenic fungi can also interfere with the vessel integrity, e.g., by tissue degradation or by formation of occlusion [27,69,70]. Since wounds are the main gate for esca-associated pathogens to enter the vascular system, the risk of infection should decrease with reduced pruning measures on the trunk [1,71,72]. Therefore, the inner-wood integrity of minimally and intensively pruned vines was examined in this study by means of white rot and necrosis in the trunk. Regarding white rot, which is mainly caused by the basidiomycete *Fomitiporia* spp., no significant differences were found between the VSP and SMPH vines irrespective of the esca status. Nevertheless, the data demonstrate that the occurrence of external esca symptoms is consistent with the extent of white rot in the trunk, since white-rot phenomena were significantly increased in symptomatic vines. Therefore, external symptoms may be positively correlated with the presence of white-rot fungi such as *Fomitiporia* spp., which is in accordance with other observations [21,73,74,75,76,77]. Previous results of reducing external esca symptoms either by trunk surgery or by use of sodium arsenite support this assumption; after treatment, the relative abundance of *F. mediterranea* in the trunk considerably decreased and external symptoms disappeared [78,79]. For wood necrosis, alone or in combination with white rot, the proportion was higher in the VSP vines than in the SMPH vines, at least in the ‘Dornfelder’ vineyard and for esca-asymptomatic vines. This is in line with the results of Travadon et al. [55], who found more necrosis in the trunks of spur-pruned vines compared to minimally pruned vines, all of which were negative for external symptoms. Intensive pruning with its higher incidence of wounds seems to increase the risk of fungal infection of the trunk, leading to inner-wood necrosis [1,80,81]. In the ‘Müller-Thurgau’ vineyard, the conversion to minimal pruning probably came too late; fungal pathogens had 24 years to enter the vascular system through one of the numerous pruning wounds and to establish themselves inside the trunk. This could be the reason why the ratio of necrosis is the same for both pruning systems in this vineyard.

A comparison of the fungal diversity in the trunk of symptomatic vines resulted in no differences between intensive and minimal pruning. With more pruning wounds, a higher diversity was expected for intensively pruned vines than for minimally pruned vines due to an increased infection rate. An explanation could be that the grapevine community in the trunk is mainly ruled by a few dominating fungi that impede the growth of other microbes [82,83]. In this study, the mycobiome in the wood samples from grapevine trunks was mainly dominated by four genera, all of which were associated with GTDs: *Diplodia*, *Eutypa*, *Fomitiporia*, *Phaeomoniella* [2]. This is not surprising given the fact that all of the sampled trunks were from vines showing external symptoms. In addition, these four fungal taxa are the most isolated fungi from necrotic and/or white-rot wood [1,9,21,22,35,84,85,86,87]. Since symptomatic wood was less abundant in esca-negative and minimally pruned ‘Dornfelder’ vines, a decline in GTD-associated fungi, especially of *Fomitiporia* spp., is expected in these trunks [78,88].

The method (metabarcoding) for the mycobiome analysis as applied in the present work has some limitations, since the classification of some OTUs at the species level based on ITS sequences alone is often not possible. Nevertheless, the species assignment can often be assumed by the isolation frequencies according to previous works. For instance, the two esca pathogens *Fomitiporia mediterranea* and *Phaeomoniella chlamydospora* are the most isolated fungi from their genus in the context of the mycobiome in German grapevine wood [21]. While the former is preferably found in vines showing external symptoms, the latter can also be abundant in externally asymptomatic vines [84,88]. Regarding *Eutypa* spp., *E. lata* is the most common representative of this genus in grapevine wood [86,88,89]. In studies focusing on the mycobiome in grapevine wood, *Diplodia seriata* is one of the most frequent fungi, irrespective of sample age or health status [75,84,86,88]. Even tough *D. seriata* is highly abundant in grapevine wood, it expresses a low pathogenicity against grapevine compared to other botryosphaeriaceous fungi [90,91,92]. Therefore, its ecological role as a pathogen or saprophyte in the grapevine microbiome is still under discussion.

Besides the four dominating fungal genera mentioned above, further GTD-associated genera were detected, although with reduced abundance. The genera *Cadophora* spp. and *Phaeoacremonium* spp. are well-known grapevine pathogens that are mainly associated with Petri disease, with *C. luteo-olivacea* and *P. minimum* being the most prevalent [93,94,95,96]. In addition, members of the genus *Diaporthe* spp., anamorph *Phomopsis* spp., were frequently observed in our study. These fungi, primarily *Diaporthe ampelina*, are the causal agent of Phomopsis dieback, but also cause Phomopsis cane and leaf spot [97,98,99]. The basidiomyceteous fungus *Phellinopsis* sp. dominated one particular sample of intensively pruned Dornfelder vine. Representatives of this genus are known to cause wood decay in forests and gardens [100,101]. *Kalmusia* spp. were recently shown to be associated with decline (*K. variispora*) and vascular necrosis (*K. longispora*) of *V. vinifera* [30,102]. Further taxa that were identified at genus level only in our study are *Seimatosporium*, *Neofabrea*, and *Truncatella*; related species known as pathogens on grapevine are *S. vitis*, *N. kienholzii*, *T. angustata* [29,103,104,105,106,107,108]. Apart from the above pathogens, a fungal genus with strong potential for biological control against GTD fungi, i.e., *Clonostachys*, was found [109,110].

## 5. Conclusions

Minimal-pruning schemes may lead to a reduction in esca. This observation is probably linked to the integrity of the vascular system. By avoiding pruning wounds, the plant has no need to form new shoots on the trunk. Therefore, the blocking of former sap routes and restructuring of the vessel system in the trunk is not necessary, which reduces the risk of sap-flow disturbance and occlusion. In addition, fewer pruning wounds on the trunk decrease the risk of infection by GTD-associated fungal pathogens, which could also interfere with the vessel integrity. In conclusion, keeping the main vascular system intact by minimal pruning reduces the occurrence of external esca symptoms. Nevertheless, the timing of pruning conversion, from intensive to minimal pruning, seems to be a critical factor, as seen for the ‘Müller-Thurgau’ vineyard. If pruning conversion takes place in older vineyards (about >10 years), the damage to the vascular system by vessel restructuring and pathogenic fungi may be too advanced and symptom expression is more likely. However, since only two vineyards were compared in this study, and due to the complexity of the disease, with several influencing factors (e.g., cultivar, age, location, climate, microbiome) involved, more research efforts need to be put towards this topic in order to verify these findings.

## Figures and Tables

**Table 1:** Properties of studied vineyards.

Cultivar	Location	Berry Color	Rootstock	Year of Planting	Training System	Year of Conversion to SMPH	Plant Protection	Plants
‘Dornfelder’	49°52′15.3″ N 8°14′03.3″ E	red	5BB	2003	SMPH	2013	conventional	2266
					SMPH	2020	conventional	726
					VSP		conventional	1403
‘Müller-Thurgau’	49°51′40.3″ N 7°50′35.7″ E	white	SO4	1984	SMPH	2008	organic	357
					VSP		organic	316

**Table 2:** Selected grapevine-trunk properties of VSP- and SMPH-trained grapevines in the two studied vineyards (‘Dornfelder’ and ‘Müller-Thurgau’). Asterisks indicate significant differences according to Student’s *t*-test (* *p* < 0.05, ** *p* < 0.001, n.s. = not significant).

	‘Dornfelder’			‘Müller-Thurgau’		
Grapevine Property	VSP	SMPH	sig.	VSP	SMPH	sig.
number of pruning wounds on trunk	17.3 ± 2.9 (*n* = 20)	9.3 ± 2.8 (*n* = 20)	**	23.0 ± 6.3 (*n* = 20)	13.3 ± 3.4 (*n* = 20)	**
pruning wound size [mm^2^]	16.1 ± 5.7 (*n* = 164)	16.2 ± 6.4 (*n* = 89)	n.s.	19.4 ± 10.3 (*n* = 178)	18.2 ± 9.0 (*n* = 133)	n.s.
trunk height [cm]	73.8 ± 5.6 (*n* = 20)	74.5 ± 3.4 (*n* = 20)	n.s.	83.4 ± 7.6 (*n* = 20)	78.6 ± 6.0 (*n* = 20)	n.s.
circumference trunk head [cm]	28.4 ± 5.0 (*n* = 20)	23.8 ± 3.5 (*n* = 20)	*	31.6 ± 5.1 (*n* = 20)	27.7 ± 4.9 (*n* = 20)	*
circumference trunk center [cm]	15.9 ± 1.6 (*n* = 20)	15.4 ± 1.2 (*n* = 20)	n.s.	17.0 ± 1.6 (*n* = 20)	16.3 ± 3.0 (*n* = 20)	n.s.

## Data Availability

Data collected in this study are available from the corresponding author upon request.
